# Prenatal diagnosis of fetomaternal hemorrhage by a novel hydrogel fluoroimmunoassay that accurately quantifies fetal haemoglobin

**DOI:** 10.3389/fbioe.2023.1194704

**Published:** 2023-06-06

**Authors:** Xinyang Li, Moli Yin, Hongmei Wang, Shengbao Duan, Huiyan Wang, Yong Li, Tiemei Liu

**Affiliations:** ^1^ Blood Transfusion Department, China-Japan Union Hospital of Jilin University, Changchun, Jilin, China; ^2^ Jilin Collaborative Innovation Center for Antibody Engineering, Jilin Medical University, Jilin City, Jilin, China; ^3^ CAS Key Lab of Bio-Medical Diagnostics, Suzhou Institute of Biomedical Engineering and Technology, Chinese Academy of Sciences, Suzhou, Jiangsu, China

**Keywords:** fluoroimmunoassay, adult haemoglobin, hydrogel medium, fetal haemoglobin, fetomaternal hemorrhage

## Abstract

**Objective:** Fetomaternal hemorrhage (FMH) is an alloimmunization resulting caused by the incompatibility between fetal and maternal blood. For the prevention of newborn haemolytic disease (HDN), it is crucial to quantify the amount of fetomaternal hemorrhage. However, the classical Kleihauer–Betke test (K-B test) for detecting fetomaternal hemorrhage is limited by experimental tools and conditions and is not suitable for routine clinical use. Consequently, the method of prenatal diagnosis of fetomaternal hemorrhage applicable to the clinic is a topic worthy of further study. Therefore, it is worthwhile to further investigation on the clinically applicable prenatal diagnosis method for fetomaternal hemorrhage.

**Methods:** This experiment demonstrates hydrogel’s ability to separate sensitized red blood cells from soluble antibodies. Using flow cytometry the fluorescence values of sensitized red blood cells and fluorophore-labeled antibodies were measured, and the testing steps for the detection products of a novel technology were determined. The properties of a hydrogel fluoroimmunoassay were evaluated by distinguishing between the amounts of fetal and adult haemoglobin. The precision of this technology is evaluated using the Kleihauer–Betke test as a comparison.

**Results:** This experiment compared the detection of haemoglobin fluorescence in adults (*n* = 2) and fetuses (*n* = 6). At the same time, the fluorescence intensity of different fetal haemoglobin (HbF) in adult haemoglobin (HbA) was calculated. The fluorescence value is 1.6% when the fetal hemoglobin concentration is 0.1%.

**Conclusion:** The novel hydrogel fluoroimmunoassay can accurately determine the fluorescence intensity by flow cytometry to differentiate fetal haemoglobin from adult haemoglobin, quantitatively prenatally diagnose fetal haemoglobin, address the incompatibility between fetal and maternal blood types, and prevent alloimmunization.

## Introduction

Evaluation of fetal red blood cells (RBCs) in the peripheral blood of pregnant women is used to detect fetomaternal hemorrhage (FMH) (Minuk et al., 2020). In order to address the incompatibility of fetal and maternal blood, it is essential to accurately quantify FMH. The number of fetal RBCs transferred to the mother determines the amount of immunoglobulin administered to prevent future Rhesus D (Rh D) alloimmunization (Glazebrook et al., 2020; Ayenew, 2021).

Fetomaternal hemorrhage is the entry of fetal red blood cells (RBCs) into the maternal circulation due to placental rupture or injury during puncture, trauma, abortion, or delivery. When the blood type of the fetus is incompatible with that of the mother, antibodies against fetal RBCs are produced in the mother. These antibodies pass through the placenta and cause the destruction of fetal RBCs (Troia et al., 2019; Hookins and Vatsayan, 2020; Monteiro et al., 2021). 16 weeks is the earliest placenta rupture, so pregnant women at 16 weeks are at risk for FMH (Akorsu et al., 2019; Athiel et al., 2020). David M conducts the Kleihauer–Betke test (K–B test) and concludes that 22.5% of Rh D-negative pregnant women carry Rh D-positive fetuses, resulting in FMH (David et al., 2004). When D-negative pregnant women have D-positive fetal RBCs, the fetal RBCs stimulate the mother to produce anti-D IgG antibody, which can enter the fetus through the placenta and produce alloimmunity (Tneh et al., 2021). Additionally, anti-Kell, anti-E, and anti-C IgG antibodies may also induce allogeneic immunity via the placenta (Erhabor et al., 2020). Alloimmunization following FMH can cause anemia, edema, jaundice, and even death in fetuses and newborns (Christino Luiz et al., 2019; Fan et al., 2021). Statistics indicate that fetal mortality due to FMH can reach 10% (Bowman, 2010). Failure to detect FMH early and treat with anti-D immunoglobulin are the leading causes of perinatal fetal morbidity and mortality (Carr et al., 2022). Therefore, prenatal diagnosis and quantification of FMH can ensure that high-risk pregnant women receive appropriate treatment and the care of experienced obstetricians. Accurate perinatal quantification of FMH and corresponding treatment are the key to addressing the incompatibility of fetal and maternal blood types and are essential for the management of obstetrics and blood transfusion departments (Pahuja et al., 2011).

The purpose of the Kleihauer–Betke test, also known as the acid elution test, is to differentiate fetal RBCs from adult RBCs (Kim and Makar, 2012) based on the difference in acid-resistance between fetal haemoglobin (HbF) and adult haemoglobin (HbA). After acid treatment, fetal RBCs appear pink and adult RBCs appear “ghost-like.” The difficulty of manually counting is the primary reason why the K-B test has not yet been implemented in the clinic (Lemaitre et al., 2020; Ficarola et al., 2022). Workers need to quickly distinguish fetal RBCs from adult RBCs according to their colour, size and texture. Usually, the staff need to count 2,000 RBCs within 20 min, and the slide contains neutrophils, overlapping cells and impurities, which makes it more difficult and subjective for the staff to count. Therefore, high labour intensity, strong subjectivity and poor accuracy have become the shortcomings of K-B test (Zhang et al., 2021). Melanie C compared the K-B test to the flow cytometry test and discovered that the K-B test yielded positive results, whereas the flow cytometry test yielded negative results. This circumstance constitutes 94% (Audette et al., 2022).

Hydrogel fluoroimmunoassay combines accurate flow cytometry (FCM) with high-sensitivity microcolumn gel technology, and further develops hydrogel immunoagglutination test into hydrogel fluoroimmunoassay technology, which improves the sensitivity and accuracy of detection technology and can be mass-produced. This technology uses a medium (1.14 g m^-1^) with a higher density than the majority of cells, i.e., the hydrogel medium’s separation properties (Wang et al., 2015). The hydrogel medium is in a flowing state at room temperature. Under the influence of centrifugal force, high-density RBCs and low-density small molecule antibodies can be directly separated, and then complicated steps such as washing can be omitted, making the procedure simple, quick, and convenient (Wu et al., 2022). The hydrogel fluoroimmunoassay uses the combination of sensitized indicator RBCs and fluorescently labelled anti-HbF antibody, and determines the fluorescence value by flow cytometry. This method is accurate and reliable in determining the quantity of fetal RBCs. Due to the fact that this method can distinguish HbF from adult haemoglobin, it can be used to detect fetal hemorrhage prenatally. This study’s objective is to evaluate the ability of hydrogel fluoroimmunoassay to diagnose FMH at a critical level of 0.1%, which is within the standard range of clinical treatment of FMH with anti-D immunoglobulin (Simes et al., 2022) and can prevent homologous immunity caused by blood group incompatibility between fetus and mother.

## Materials and methods

### Reagents, instruments and blood samples

The anti-mouse IgG (fab specific)-FITC antibody produced in goal was acquired from Sigma (F5387). All chemical reagents were acquired from Sinopharm Chemical Reagents Co., Ltd., (China). Alexa Fluor TM 647 Carboxylic Acid, Succinimidyl Ester was purchased from Thermo Fisher scientific (United States). Flow cytometer (z2010-254, BD LSRFortessa). In our laboratory, 0.01M PBS (pH 7.2) and hydrogel were prepared. Two lymphocyte hybridoma cells produce the monoclonal antibody against HbF (15-G4, 9-F10). The sample of whole blood was supplied by China-Japan Union Hospital of Jilin University. The blood sample stored at 4°C has been approved by the China-Japan Union Hospital’s Ethics Committee (Approval number: 20220425009).

### Blood sample preparation and extraction of haemoglobin

Blood from healthy adults and umbilical cord blood from newborn fetal umbilical cords were collected and washed three times with saline. Then, added twice volume of distilled water, which is erythrocyte lysate, centrifuged at 10,000 r.p.m for 20 min, collected the supernatant, which was haemoglobin, and determined its concentration. The same concentration of fetal hemoglobin was then mixed with adult hemoglobin in volumes of 1: 1,000, 3: 1,000, and 5: 1,000 to produce fetal hemoglobin with proportions of 0.1%, 0.3%, and 0.5% in the mixed hemoglobin.

### Separation of indicator RBCs from small molecular dyes by hydrogel

Wang’s article described the use of hydrogel to separate small molecular dyes from indicator RBCs. The characteristics of methylene blue and the reaction time of the reactants differed between this experiment and Wang’s. In this experiment, methylene blue (Mw373.90,0.8%), indicator RBCs, and additional reactants were placed on top of hydrogel, incubated, and centrifuged at 285 g for 3 min.

### The preparation of indicator RBCs

Randomly collected “O” type Rh-positive RBCs, thoroughly washed them, treated them with 0.5% GA-PBS solution at room temperature for 30 min, centrifuged and washed them, then added 0.5% PA-PBS solution to water bath at 37°C for 1 h, centrifuged and washed them, then added 1% PFA-PBS solution to water bath at 37°C for 5 h, and shook them every 30 min. After washing and centrifuging, a 10% suspension of RBCs was made and stored at 4°C for future use.

### The method of coating indicator RBCs with antibodies

Chromium trichloride solution (CrCl3.6H_2_O) in saline had a concentration of 1.51%. Adjust the pH to 5.0 using 1 mol/L sodium hydroxide prior to use. By using the metal cation immunoglobulin tannic acid method, aldehyde-modified RBCs were made sensitive. In other words, chromium trichloride solution was added to anti-HbF antibody diluted with acetate buffer (0.175 M pH6.4) to achieve a final concentration of 120 μg/mL. After mixing, water-bathing at 37°C for 10 min. To the anti-HbF antibody and 1 mL of hematocrit that had been incubated, 1:2,000 tannic acid solution was added immediately. It is necessary to ensure that the volume ratio of the mixed solution to the tannic acid solution is 3:1, and to incubate the solution in a 37°C water bath for 15 min. After six times of washing, 2% BSA-PBS was added to a 37°C water bath for 2 hours, and a storage suspension was subsequently prepared.

### Verification of binding of indicator cell and anti-HbF antibody

As the second antibody, sheep anti-mouse IgG FITC was used to detect mouse anti-HbF antibody. After 30 min in a 37°C water bath, the cells were washed once and centrifuged before being collected. The hematocrit was then diluted in PBS-BSA at 1%. Using FCM, the fluorescence intensity of negative and positive samples was measured.

### Method of labeling antibody with fluorescein

Thermo Fisher Scientific Company’s Alexa Fluor TM 647 Carboxylic Acid, Succinimidyl Ester reagent was used to label anti-HbF antibody with F647. Among them, the goat anti-mouse IgG antibody concentration was 16.544 mg, and the labeling time of F647 for goat anti-mouse IgG antibody was 4 h at room temperature, followed by an overnight treatment at 4°C.

### Hydrogel fluoroimmunoassay for HbF detection

**FIGURE 1:**
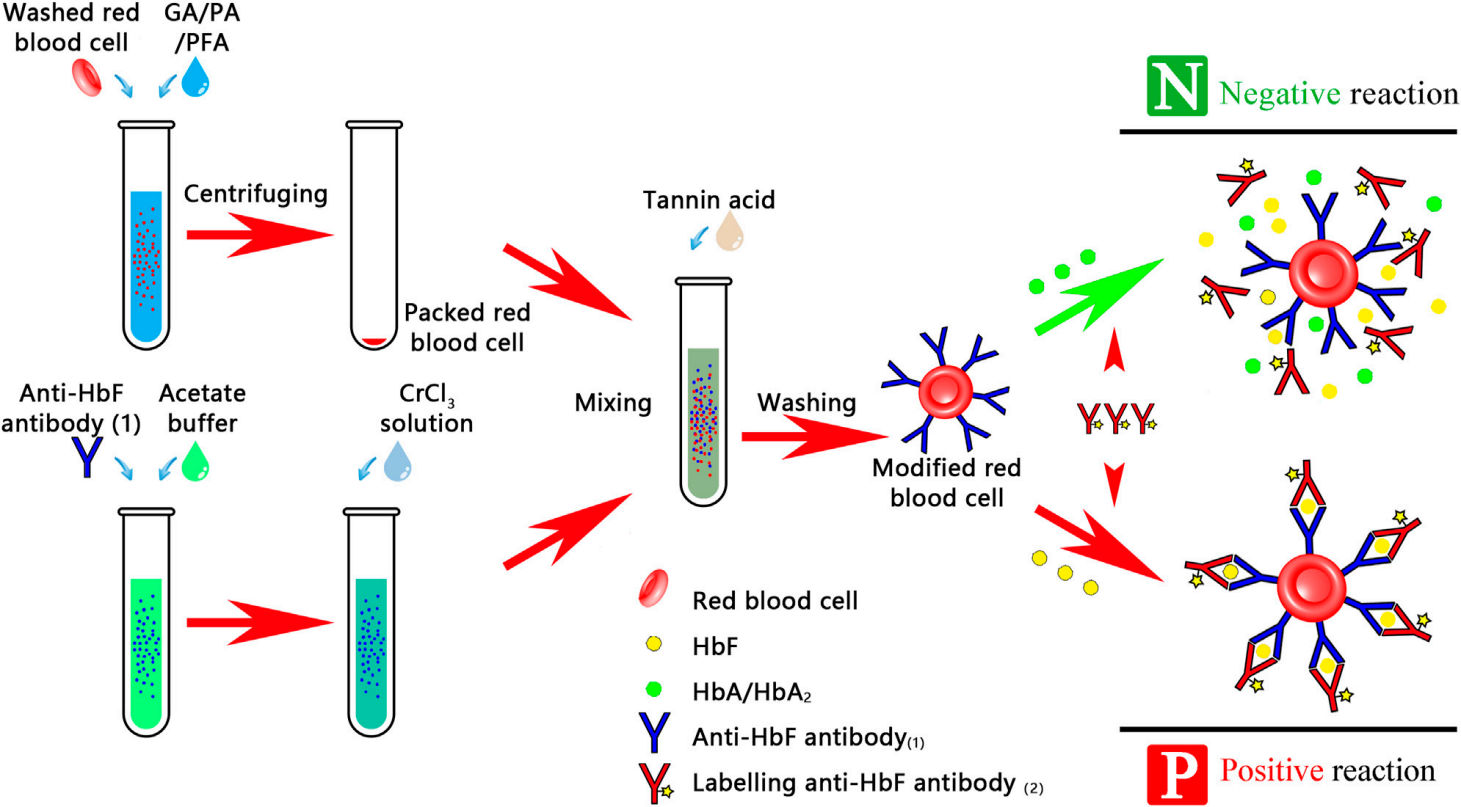
Procedure for detecting FMH using a hydrogel fluoroimmunoassay technique. This experiment was divided into two stages: the first stage was prepared by research units, which includes fluorescently labeled antibody, indicator RBCs-coated antibody, and RBC lysis; the second stage was prepared by hospital transfusion departments.

The haemoglobin (Hb), fluorescence-labeled antibody, and sensitized indicator RBCs were added to the hydrogel-filled card and centrifuged at a low speed until the immune complex in the positive result and the indicator RBCs in the negative result sank, while the fluorescence-labeled antibody remained on the hydrogel in the negative result (Figure 1).

### Analysis of fetal red blood cells in hydrogel fluoroimmunoassay

The hydrogel fluoroimmunoassay substances were detected by flow cytometry calibrated with standard fluorescent microspheres, and the fluorescence intensity of 50,000 cells was determined. The operation steps strictly adhere to the BD LSRFortessa flow cytometry instructions. The flowchart is examined with Flowjo 10.8.1. The distinction between fetal and adult red blood cells is based on the fact that their peaks are distinct (Figure 4). Even though the negative control has only one peak (0.0% fetal hemoglobin), this experiment distinguishes it using the abscissa value of 10^3^ as the boundary. Flow cytometry values with 0.1%, 0.3%, and 0.5% of fetal hemoglobin are subtracted from negative control values greater than 10^3^ according to the principle of data processing.

### Analysis of the sensitivity of the K-B test and hydrogel fluoroimmunoassay

The preceding analysis method was used to analyse and compare the K-B test. The K-B test was conducted in accordance with the AABB technical manual. The experiment involved the observation of 10,000 cells and the counting of fetal RBCs (Athiel et al., 2020). This experiment was repeated three times, and the results were statistically analyzed using GraphPad Prism 9.4.1 to determine if there was a statistically significant difference between the hydrogel fluoroimmunoassay and K-B test for detecting different fetal red blood cells produced by 0.0%, 0.1%, 0.3%, and 0.5% cord bloods.

## Results

### Effect of hydrogel medium on the separation of small molecular dyes and indicator RBCs

**FIGURE 2:**
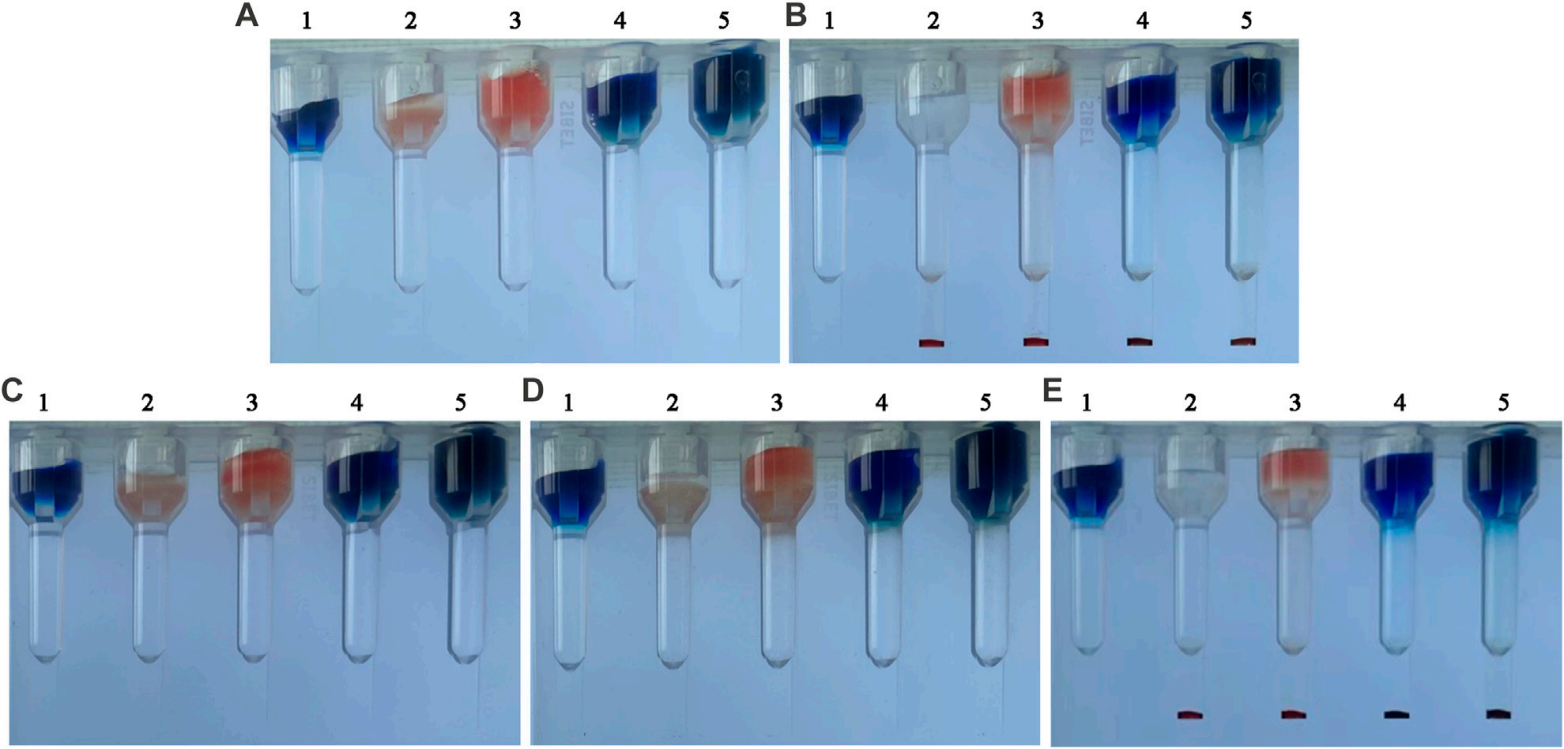
Separation results of small molecule dyes and indicator RBCs using a hydrogel medium. **(A)** before centrifugation, from left to right: (A)_1_ Methylene blue, (A)_2_ indicator RBC-coated antibodies (A)_3_ immune complex components, (A)_4_ indicator RBC-coated antibodies and methylene blue, and **(A)**_5_ immune complex components and methylene blue. **(B)** after centrifugation. **(C)** before incubation. **(D)** 1-h incubation. **(E)** centrifugation immediately following incubation.

To demonstrate that the hydrogel is capable of separating indicator RBCs from small molecular proteins not bound to RBCs, we compared the distribution of small molecular substances, RBCs, and immune complexes in the hydrogel medium under different conditions. The substances with the higher density sank to the bottom of the hydrogel medium, whereas the substances with the lower density remained on top of the hydrogel. Figure 2D indicated that 1 h of incubation had no effect on the diffusion of small molecules or RBCs. In Figure 2, (A)_1_ and 2 (B)_1_ remained unchanged, indicating that methylene blue was not centrifuged to the card’s bottom. Red indicator red blood cells coated with an antibody were added to the top of the 2 (A)_2_ card. The top of the card in 2 (B)_2_ changed from red to white after centrifugation, and red (indicator RBC-coated antibodies) appeared at the bottom of the card, indicating that the antibody-coated indicator red blood cells were centrifuged to the bottom of the card. The 2 (A)_3_ card turns red when the immune complex (indicator RBC-coated antibodies, fluorescently labeled antibodies, and excessive hemoglobin to be detected) is added. The immune complex formed by indicator RBC-coated antibodies—HbF—fluorescently labeled antibodies is centrifuged to the bottom of the card to form red, while hemoglobin at the top of the card is not bound to form an immune complex and is therefore still red. The experimental findings were consistent with Wang’s perspective (Wang et al., 2015). The results demonstrated that hydrogel medium could indeed separate soluble antigens, soluble antibodies, granular substances, and other macromolecules. The centrifugal force of RBCs and small molecules sinking in this experiment was 285 g.

### Analysis of the binding of indicator cells to antibodies

**FIGURE 3:**
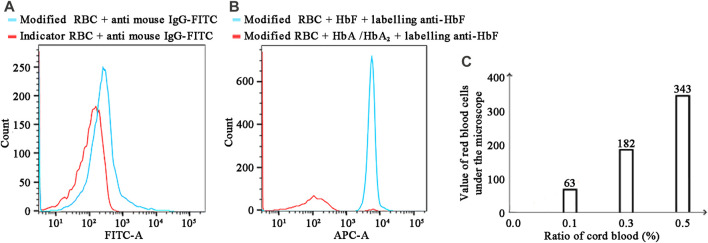
**(A)** Detection results of bound antibodies using FITC-conjugated goat anti-mouse IgG. **(B)** FCM detection of hemoglobin using Alexa Fluor™ 647-conjugated anti-HbF. **(C)** The K–B test results for various cord blood ratios in the mode of FMH.

Figure 3A's objective is to determine if the indicator cells were bound to anti-HbF antibody. In comparison to the red line (negative sample), the light blue line (positive sample) is clearly shifted to the right, and the fluorescence value is clearly elevated. The outcomes demonstrated that the anti-HbF antibody was successfully coupled with indicator RBCs, which could be used in the subsequent experiment.

### Immune complex detection with fluorescent-labeled antibodies

**FIGURE 4:**
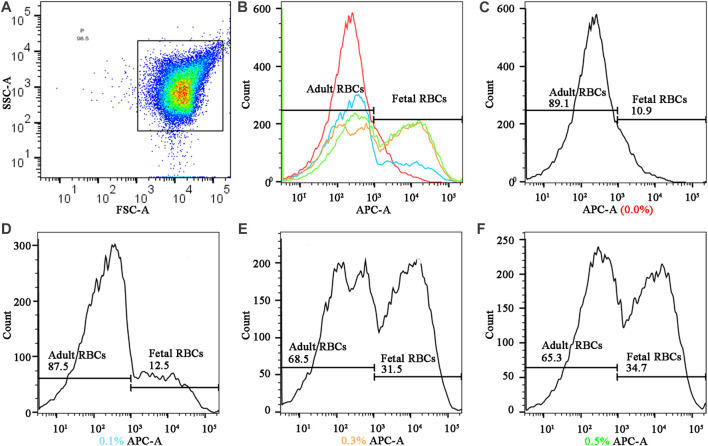
Fluorescence quantification results of different cord blood ratios in the mode of FMH by hydrogel fluoroimmunoassay technique **(A)** Gating strategy: The fetal RBCs gate was defined using a positive control fetal control and a negative adult control. **(B)** the summarized figures obtained by superimposing the **(C) (D)**, **(E)**, and **(F)** figures, differentiated by line colors; **(C)**, **(D) (E)**, and **(F)**, and f represented the fluorescence intensities of various proportions of fetal hemoglobin in mixed hemoglobin, respectively (0.0%, 0.1%, 0.3%, and 0.5%).

**FIGURE 5:**
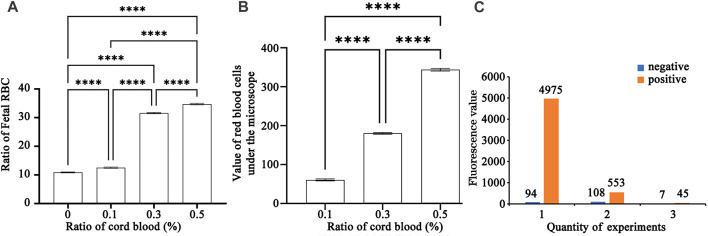
**(A)** A bar chart of repeated experiments using hydrogel fluoroimmunoassay to detect various cord blood ratios. ✳: The magnitude of differences between the various percentages of umbilical cord blood (0.0%, 0.1%, 0.3%, and 0.5%).**(B)** A bar chart of repeated experiments using K-B test to detect various cord blood ratios. ✳: The magnitude of differences between the various percentages of umbilical cord blood (0.1%, 0.3%, and 0.5%).**(C)** Diagram of negative and positive control in a repeated experiment.

Figure 3B demonstrated that F647-labeled antibody and antibody modification indicated that RBCs could indeed recognise HbF specifically, indicating that the experimental steps had been established successfully. Compared to the red line (negative sample), the light blue line (positive sample) was clearly shifted to the right, and the fluorescence value was clearly increased, indicating that this experiment was able to distinguish between fetal and HbA. As depicted in Figure 5C, this experiment was repeated three times with negative and positive controls. The Hb concentration used in this experiment was 0.608 mg/mL (0.60–0.62 mg/mL). In the traditional FCM detection method, the incubated reactants were required to be washed a minimum of three times; if the washing times were insufficient, the false positive rate would increase, and if they were excessive, fetal RBCs would be lost. It had been reported in the literature that washing times should be minimised as much as possible when detecting fetal bleeding with FCM (Wiley Online Library, 1999). Figure 3B depicted the detection results obtained after a single washing, while Figure 4depicted the separation results obtained using a hydrogel medium. It was discovered that the fluorescence intensity of washing once was demonstrably higher than that of hydrogel separation, indicating that the separation effect of hydrogel was demonstrably more effective than that of washing once. Consequently, the use of hydrogel medium can reduce washing steps, save time, and ensure the accuracy of experimental results.

### Hydrogel fluoroimmunoassay and K-B test sensitivity

Figure 4 depicts the detection of mixed haemoglobin in various proportions (the ratio of HbF to HbA) via hydrogel fluoroimmunoassay. In this experiment, 50,000 cells were counted. Figure 4B depicted the global distribution of HbF concentrations of 0.0%, 0.1%, 0.3%, and 0.5%. The fluorescence intensity increased as the ratio of fetal RBCs increased. As shown in the image, the negative result (0.0%) had a peak that represented HbA; there were two peaks with varying proportions of HbF (0.1%, 0.3%, and 0.5%). HbA was represented by the peak on the left, while HbF was represented by the peak on the right. As the fetal RBC ratio increased, the height of the left peak decreased, while the height of the right peak increased. Positive APC-A values for 0.1% HbF were found to be 1.6%, 0.3% HbF was 20.6%, and 0.5% HbF was 23.8%. As the fetal RBC ratio increased, the APC-A positive value increased dramatically. Figures 4C–F represented sub-graphs for HbF concentrations of 0.0%, 0.1%, 0.3%, and 0.5%, respectively. Figure 3C depicted the outcomes of the K-B test on blood samples with varying proportions. Using electron microscopy, 10,000 cells were tallied; Figure 3C displays an upward trend in the proportion of fetal RBCs within adult RBCs. The K-B test and the hydrogel fluoroimmunoassay exhibited a strong correlation. Both were able to reliably and accurately detect the amount of FMH, and their sensitivity can reach 0.1%, which is in line with the clinical treatment standard for 2 mL of FMH. On the basis of these results, a critical concentration of 0.1% is determined. The conclusion reached by Davis is consistent with this one (Davis et al., 2010). Figure 5 was a bar graph of repeated hydrogel fluoroimmunoassay and K-B tests for different hemoglobin concentrations. *p* < 0.0001 indicated that the fluorescence values of 0.0%, 0.1%, 0.3%, and 0.5% of fetal hemoglobin detected by hydrogel fluoroimmunoassay were statistically significant. Figure 5B demonstrates that the K-B test was statistically significant for detecting fetal red blood cells in 0.1%, 0.3%, and 0.5% of mixed blood (*p* < 0.0001).

## Discussion

In this study, fetal hemorrhage was diagnosed quantitatively using the hydrogel fluoroimmunoassay. It is believed that this is the first time the combination of hydrogel medium and fluoroimmunoassay have been combined to diagnose FMH. This experiment also assessed the precision of FMH and compared it to the K-B test. By employing this technique, the risk of alloimmunization due to maternal and fetal blood group incompatibility can be reduced. Consequently, hydrogel fluoroimmunoassay technology may be advantageous for the diagnosis and prevention of FMH. Evaluation of FMH using hydrogel fluoroimmunoassay required minimal blood (approximately 10 μL), minimal dependence on manual operation.

Inability to distinguish between HbF and F cells is a common source of false-positive results in K-B tests (Flynn et al., 2022). F cells refer to normal RBCs (Alter, 1984; Fabry et al., 2001; Corcoran et al., 2014) that contain a small amount of HbF (1%–2% in adults) (Cormack et al., 2019). Nonetheless, certain inherited or acquired haemoglobin disorders, such as sickle cell anaemia and -thalassemia, result in a substantial increase in HbF levels. In addition, F cells will physiologically increase during pregnancy. Hemoglobin in F cells and fetal RBCs has different antigen specificity to support the distinct differentiation of fetal RBCs and adult RBCs, resulting in different fluorescence intensities (Cardoso et al., 2019; Stanic et al., 2020). The anti-HbF antibody used in this experiment is a specific antibody against two distinct epitopes of HbF, and the adult HbF sample, which is the negative control, is used as the negative control. The fluorescence value of fetal hemorrhage minus the fluorescence value of the negative control is the final fluorescence result produced by different proportions of HbF in order to avoid the influence of the increase of HbF in F cells on the detection results (Hsia et al., 2019). In addition, the results were confirmed by repeating experiments with three adult erythrocytes and three fetal erythrocytes (Figure 5C). Therefore, the anti-fetal haemoglobin antibody fluorescence value calculated by the hydrogel fluoroimmunoassay will not prompt patients to take additional anti-D immunoglobulin. In addition, compared to the article by Agaylan, A. and Kumpel, B., this technique has the advantages of not requiring washing, high efficiency, loss-free separation of antigen-antibody complexes, higher sensitivity, simple and rapid operation, reduced fibrinogen interference, and elimination of false positives (Agaylan et al., 2007; Kumpel et al., 2014). FCM is more advantageous in this study for detecting fetal RBCs and F cells in blood samples, consistent with Marcella R’s report (Cardoso et al., 2019). In this experiment, the centrifugal force that causes red blood cells sensitized with anti-HbF to sink is 285 g. The article by Wang demonstrates that the minimum centrifugal force required for red blood cells to sink is less than 100 g, whereas the minimum centrifugal force required for proteins to sink is 3,000 g. In this experiment, the centrifugal force is greater than in Wang’s article. The primary cause may be that the red blood cells in this experiment have been sensitized by anti-HbF and their molecular weight is greater than that of red blood cells.

Preventing and treating fetal hemorrhage requires not only prenatal and perinatal diagnosis, but also monitoring of pregnant women and newborns after delivery. Hydrogel fluoroimmunoassay can monitor the concentration of HbF, which can determine the drug dosage and disease severity (Garner et al., 2000; Franco et al., 2006). This method is expected to be used to diagnose various obstetric diseases, including placental intervillous thrombosis, due to its simplicity, speed, and efficacy (Sukhanova et al., 2022).

Nonetheless, this experiment has some restrictions. In the experiment, the sensitized RBCs at the bottom of the hydrogel medium are extracted using a needle and then detected using FCM. Due to the complexity of this step and the high cost of FCM, hospitals with small sample sizes are unable to run FCM continuously (Kim and Makar, 2012). The development of a small analyzer that can quantitatively detect the fluorescence intensity at the bottom of a hydrogel medium warrants further study.

## Data Availability

The raw data supporting the conclusions of this article will be made available by the authors, without undue reservation.
